# Differentiation of high grade glioma and solitary brain metastases by measuring relative cerebral blood volume and fractional anisotropy: a systematic review and meta-analysis of MRI diagnostic test accuracy studies

**DOI:** 10.1259/bjr.20220052

**Published:** 2022-12-08

**Authors:** Fioni Fioni, Song Jia Chen, I Nyoman Ehrich Lister, Abdelrahman Atef Ghalwash, Ma Zhan Long

**Affiliations:** Department of Radiology, Nanjing Medical University, first affiliated hospital (Jiangsu Provincial People’s Hospital), Jiangsu, China; Department of Radiology, Nanjing Medical University, first affiliated hospital (Jiangsu Provincial People’s Hospital), Jiangsu, China; Medicine, Universitas Prima Indonesia and Royal Prima Hospital, Medan, North Sumatera, Indoneisa; Department of Medicine, Kafrelsheikh University, Kafrelsheikh, Egypt; Department of Radiology, Nanjing Medical University, first affiliated hospital (Jiangsu Provincial People’s Hospital), Jiangsu, China

## Abstract

**Objective:**

This study aims to research the efficacy of MRI (I) for differentiating high-grade glioma (HGG) (P) with solitary brain metastasis (SBM) (C) by creating a combination of relative cerebral blood volume (rCBV) (O) and fractional anisotropy (FA) (O) in patients with intracerebral tumors.

**Methods:**

Searches were conducted on September 2021 with no publication date restriction, using an electronic search for related articles published in English, from PubMed (1994 to September 2021), Scopus (1977 to September 2021), Web of Science (1985 to September 2021), and Cochrane (1997 to September 2021). A total of 1056 studies were found, with 23 used for qualitative and quantitative data synthesis. Inclusion criteria were: patients diagnosed with HGG and SBM without age, sex, or race restriction; MRI examination of rCBV and FA; reliable histopathological diagnostic method as the gold-standard for all conditions of interest; observational and clinical studies. Newcastle-Ottawa quality assessment Scale (NOS) and Cochrane risk of bias tool (ROB) for observational and clinical trial studies were managed to appraise the quality of individual studies included. Data extraction results were managed using Mendeley and Excel, pooling data synthesis was completed using the Review Manager 5.4 software with random effect model to discriminate HGG and SBM, and divided into four subgroups.

**Results:**

There were 23 studies included with a total sample size of 597 HGG patients and 373 control groups/SBM. The analysis was categorized into four subgroups: (1) the subgroup with rCBV values in the central area of the tumor/intratumoral (399 HGG and 232 SBM) shows that HGG patients are not significantly different from SBM/controls group (SMD [95% CI] = −0.27 [-0.66, 0.13]), 2) the subgroup with rCBV values in the peritumoral area (452 HGG and 274 SBM) shows that HGG patients are significantly higher than SBM (SMD [95% CI] =  −1.23 [-1.45 to -1.01]), (3) the subgroup with FA values in the central area of the tumor (249 HGG and 156 SBM) shows that HGG patients are significantly higher than SBM (SMD [95% CI] = - 0.44 [-0.84,–0.04]), furthermore (4) the subgroup with FA values in the peritumoral area (261 HGG and 168 SBM) shows that the HGG patients are significantly higher than the SBM (SMD [95% CI] = −0.59 [-1.02,–0.16]).

**Conclusion:**

Combining rCBV and FA measurements in the peritumoral region and FA in the intratumoral region increase the accuracy of MRI examination to differentiate between HGG and SBM patients effectively. Confidence in the accuracy of our results may be influenced by major interstudy heterogeneity. Whereas the I^2^ for the rCBV in the intratumoral subgroup was 80%, I^2^ for the rCBV in the peritumoral subgroup was 39%, and I^2^ for the FA in the intratumoral subgroup was 69%, and I^2^ for the FA in the peritumoral subgroup was 74%. The predefined accurate search criteria, and precise selection and evaluation of methodological quality for included studies, strengthen this study

Our study has no funder, no conflict of interest, and followed an established PROSPERO protocol (ID: CRD42021279106).

**Advances in knowledge:**

The combination of rCBV and FA measurements’ results is promising in differentiating HGG and SBM.

## Introduction

^1^WHO 2007 classified the two most usual brain neoplasms in adults as high-grade gliomas (HGGs) and brain metastasis.^1^ The most usual complication of systemic tumors is brain metastasis, with half of all cases being solitary at the time of diagnosis.^2^ Astrocytoma, anaplastic astrocytoma, and glioblastoma are examples of astrocytic tumors/glioma.^3^ Because solitary brain metastasis (SBM) and HGG have different treatment planning, follow-up, prognosis, tumor stage, and clinical outcomes, it is crucial to distinct GBM from SBM in clinical practice.^4^ On standard MR imaging, distinguishing between a solitary metastatic tumor and HGG is difficult because they have similar signal intensity features, imaging features, and contrast enhancement forms, such as severe edema and ring enhancement, making clinical treatment difficult.^5^

Glioblastoma is an infiltrative malignancy that spreads to surrounding tissue and white matter pathways. Microscopically, it spreads many centimeters beyond the imaging enhancing zone known as infiltrative edema.^5^ In contrast, metastasis expands outwards, displacing neighboring tissues but without creating infiltrative edema. According to this view, the most effective approach for precisely defining the lesion would be to focus on and evaluate peritumoral features.^6,7^

The peritumoral region is described as an area outside/surrounding the solid section of the tumor,^8^ while the intratumoral zone is described as the area within the solid component of the tumor itself.^9^

GBs and metastatic brain tumors are recognized to produce angiogenesis, which results in raised perfusion.^10^ Due to its capacity to identify angiogenesis changes and measure microenvironmental changes at the capillary stage/vascularity,^11^ PWI has been proven in multiple studies to be a possible tool for differentiating GBM from SBM.^12^ As a result, several studies have turned to perfusion MR imaging to distinguish GB from brain metastases.^13–16^ DSC may now be utilized as a diagnostic tool^16,17^ by calculating the rCBV based on tumor infiltration in the peritumoral region and providing a quantitative assessment of neovascularization.^7^

Diffusion tensor imaging (DTI) (DWI-MRI) is a quite new method and one of the techniques that may correctly redirect the microstructure of tissues by detecting tissues’ diffusion of water molecules.^18^ The quantity of directed water diffusion in the brain parenchyma, and directionality in the brain, are measured using fractional anisotropy (FA). FA diffusion measures the tensors’ related values, which can be linked to anisotropic diffusion, the direction in which water flows.^19^ High FA values should be found in white matter tracts that travel by a single axis, whereas low FA values should be found in free water regions like ventricles.^20^ It is assumed to be a trait related to the architecture and fiber integrity of white matter in the brain parenchyma.^21^ FA decreases in wounded tissues in the general cause by the stoppage of directed water transport. Axonal architecture, vascularity, cell density, fiber tracts, and neuronal structures have all been associated with FA.^22,23^ Prior to now, HGGs and SBMs have been distinguished using DTI, with the most widely used metrics being DTI’s FA. On the other hand, conflicting findings regarding the capacity of FA to distinguish HGGs from SBMs have been reported.^9^

Since it is unclear if multimodal MRI can tell apart SBM from HGGs,^24^ according to several of these studies, combining diffusion and perfusion parameter data can help discriminate between solitary SBM and HGG.^2,25^ Several authors have recently coupled 1H-MRSI, DWI, and PWI with conventional MRI to increase its ability to differentiate solid tumors from other intratumoral or peritumoral components.^12,16^

Our research question was how is the efficacy of MRI (I) using perfusion magnetic resonance measurements of variable rCBV (O) and diffusion magnetic resonance measurements of variable FA (O) for differentiate HGG (P) with SBM (C) in patients with intracerebral tumors.

In this study, we predicted that by utilizing a combination of perfusion MR of rCBV parameters and diffusion MR of FA parameters, measurements added to the MRI protocol might improve the accuracy of differentiating between HGG and SBM. Moreover, it is something that should be taken into account regularly.

## Methods

### Study design

This systematic review and meta-analysis adhered to the PROSPERO (ID: CRD42021279106) methodology and followed the Preferred Reporting Items for Systematic Reviews and Meta-analysis (PRISMA) statement.

## Search strategy

We searched electronic engines Web of Science through Clarivate, PubMed, Scopus, and Cochrane library, to collect relevant studies. All relevant articles were searched on September 14, 2021 without any limitation on publication date and were located on search results within the databases used. A clear MeSH and ‘text word’ input, with Boolean operators, input into databases used. We excluded non-human studies, non-English, non-articles types, and non-journal, which were available by automation tools. We conduct our search by using the entries pattern as follows:

(different* OR discriminat* OR distinguish OR distinct*) AND ( glioblastoma* OR gbm OR gb OR astrocyt* OR gliom* OR gliosarcom* OR “glioblastoma multiforme” OR “multifocal glioblastoma” OR “multicentric glioblastoma” OR “grade iv astrocytoma” OR “giant cell glioblastoma”) AND ( “solitary brain metast*” OR “solitair* brain metasta*” OR “single brain metasta*” OR “neoplasm metasta*” OR “tumor metasta*” OR “cns metas*” OR “central nervous system metast*” OR tumor) AND ( “relative cerebral blood volume” OR rcbv OR “cerebral blood volume” OR “fractional anisotropy” OR “mean diffusivity”). **The preliminary search strategy is given in** Appendix A. We also manually scanned the key papers to find other relevant references.

## Selection criteria and process

### Inclusion criteria

Our systematic review inclusion criteria are as follows, studies: (1) reported only on humans; (2) conducted on populations without limitations to countries, age, sex, or race; (3) should use MRI diagnostic method for all conditions of interest; (4) reported perfusion metrics and diffusion measured in HGGs and SBMs, with mean rCBV or FA assessment obtainable for valuable results; (5) the examination way was a region of interest (ROI) analysis, with intratumoral or peritumoral areas researched; (6) study types clinical trials and observational studies; (7) the publication year has no restrictions.

## Exclusion criteria

(1) Studies were not issued in peer-reviewed, (2) studies not published in English language; (3) studies did not present the prevalence of HGG and SBM; (4) studies with control groups diagnosed with multiple brain metastasis; (5) case reports and reviews studies; (6) gray literature, not to be included.

### Study selection and data extraction

Outcomes acquired from the search strategy were evaluated for duplication with Mendeley and excel. Afterward, unnoticed duplicates were removed manually. The search results were reviewed by two authors in accordance with the inclusion and exclusion criteria to check titles and abstracts for relevancy. Two authors assessed the full text and screened the studies according to the requirements. Different opinion on study eligibility was solved through the authors’ debate.

Two authors were assigned to extract data from the selected studies. We extracted data relating to authors, study year, study design, country, population type, number of participants, available data on participant’s age, region, number of patients with HGG, number of patients with SBM, the strength of the magnetic field; a diagnostic method of MRI/analysis method, condition of participants, the outcome of studies; parameter values in peritumoral or intratumoral regions additional data on subgroups, and additional notes (Table 1, Table 2, Table 3).

**Table 1.** Extracted data of DSC metrics rCBV variable & DWI metric FA variable in the intratumoral and peritumoral regions in included studies

Study Author (Study year)	Title	Area peritumoral/ intratumoral	High- grade glioma rCBV (mean ± SD)	Solitary brain metastasis rCBV (mean ± SD)	High- grade glioma FA (mean ± SD)	Solitary brain metastasis FA (mean ± SD)	Total participants
Mao J, et al.^9^ 2020	Differentiation between high-grade gliomas and solitary brain metastases: a comparison of five diffusion-weighted MRI models	Intratumoral	NA	NA	0.33 ± 0.13	0.22 ± 0.09	41
		Peritumoral	NA	NA	0.35 ± 0.11	0.33 ± 0.15	
Kadota Y, et al. ^26^ 2020	Differentiation between glioblastoma and solitary brain metastasis using neurite orientation dispersion and density imaging	Intratumoral	NA	NA	0.145 ± 0.082	0.098 ± 0.042	15
		Peritumoral	NA	NA	0.170 ± 0.024	0.158 ± 0.065	
She D, et al. ^27^ 2019	Differentiation of glioblastoma and solitary brain metastasis by gradient of relative cerebral blood volume in the peritumoral brain zone derived from dynamic susceptibility contrast perfusion MRI	Intratumoral	NA	NA	NA	NA	43
		Peritumoral	0.73 ± 0.37	0.51 ± 0.24	NA	NA	
Mouthuy N, et al. ^28^ 2012	Multiparametric MRI to differentiate high-grade gliomas and brain metastases	Intratumoral	10.7 ± 5.3	6.63 ± 4.61	NA	NA	46
		Peritumoral	1.91 ± 1.69	0.74 ± 0.49	NA	NA	
Abdel Razek AAK, et al. ^29^ 2019	Differentiating glioblastomas from solitary brain metastases using arterial spin labeling perfusion- and diffusion tensor imaging-derived metrics	Intratumoral	NA	NA	0.14 ± 0.04	0.17 ± 0.03	36
		Peritumoral	NA	NA	0.22 ± 0.08	0.32 ± 0.07	
Svolos P, et al. ^30^ 2013	Investigating brain tumor differentiation with diffusion and perfusion metrics at 3T MRI using pattern recognition techniques	Intratumoral	7.14 ± 2.33	7.80 ± 2.61	0.148 ± 0.058	0.117 ± 0.040	71
		Peritumoral	2.67 ± 1.06	0.94 ± 0.35	0.286 ± 0.069	0.251 ± 0.048	
Blasel S, et al. ^7^ 2010	Elevated peritumoural rCBV values as a mean to differentiate metastases from high-grade gliomas	Intratumoral	NA	NA	NA	NA	52
		Peritumoral	1.17 ± 0.32	0.78 ± 0.17	NA	NA	
Aslan K, et al. ^31^ 2019	Multiparametric MRI in differentiating solitary brain metastasis from high-grade glioma: diagnostic value of the combined use of diffusion-weighted imaging, dynamic susceptibility contrast imaging, and magnetic resonance spectroscopy parameters	Intratumoral	3.63 ± 1.40	3.68 ± 1.40	NA	NA	56
		Peritumoral	1.14 ± 0.46	0.39 ± 0.18	NA	NA	
Tan Y, et al. ^32^ 2015	Differentiation of high-grade astrocytomas from solitary brain metastases: Comparing diffusion kurtosis imaging and diffusion tensor imaging	Intratumoral	NA	NA	0.21 ± 0.20	0.18 ± 0.07	51
		Peritumoral	NA	NA	0.18 ± 0.05	0.16 ± 0.03	
Tsolaki E, et al. ^25^ 2013	Automated differentiation of glioblastomas from intracranial metastases using 3T MR spectroscopic and perfusion data	Intratumoral	7.13 ± 3.17	7.73 ± 4.36	NA	NA	49
		Peritumoral	2.81 ± 1.44	1.29 ± 0.61	NA	NA	
Bauer AH, et al. ^2^ 2015	Differentiation of solitary brain metastasis from glioblastoma multiforme: a predictive multiparametric approach using combined MR diffusion and perfusion	Intratumoral	3.87 ± 1.17	2.55 ± 1.20	0.23 ± 0.04	0.11 ± 0.05	23
		Peritumoral	1.71 ± 1.21	0.94 ± 1.25	0.27 ± 0.05	0.16 ± 0.05	
Neska-Matuszewska M, et al. ^33^ 2018	Differentiation of glioblastoma multiforme, metastases and primary central nervous system lymphomas using multiparametric perfusion and diffusion MR imaging of a tumor core and a peritumoral zone-Searching for a practical approach	Intratumoral	3.10 ± 1.50	4.49 ± 3.94	NA	NA	57
		Peritumoral	1.05 ± 0.39	0.55 ± 0.13	NA	NA	
Chiang IC, et al. ^15^ 2004	Distinction between high-grade gliomas and solitary metastases using peritumoral 3 T magnetic resonance spectroscopy, diffusion, and perfusion imagings	Intratumoral	0.09 ± 0.05	0.22 ± 0.23	NA	NA	26
		Peritumoral	2.33 ± 1.61	0.84 ± 0.33	NA	NA	
Zhang H., et al. ^34^ 2009	Differentiation between supratentorial single brain metastases and high grade astrocytic tumors: An evaluation of different DSC MRI measurements	Intratumoral	6.00 ± 2.17	2.75 ± 1.72	NA	NA	53
		Peritumoral	1.77 ± 1.19	1.05 ± 0.53	NA	NA	
Svolos P., et al. ^35^ 2013	Classification methods for the differentiation of atypical meningiomas using diffusion and perfusion techniques at 3 T MRI	Intratumoral	10.95 ± 6.55	8.92 ± 3.61	0.140 ± 0.052	0.116 ± 0.040	42
		Peritumoral	1.81 ± 0.59	1.23 ± 0.38	0.291 ± 0.085	0.279 ± 0.046	
Lu S., et al. ^36^ 2003	Peritumoral diffusion tensor imaging of high-grade gliomas and metastatic brain tumors	Intratumoral	NA	NA	NA	NA	24
		Peritumoral	NA	NA	0.248 ± 0.063	0.181 ± 0.041	
Cindil E., et al. ^37^ 2021	Validation of combined use of DWI and percentage signal recovery-optimized protocol of DSC-MRI in differentiation of high-grade glioma, metastasis, and lymphoma	Intratumoral	4.01 ± 2.51	4.25 ± 3.05	NA	NA	84
		Peritumoral	1.61 ± 0.99	0.77 ± 0.31	NA	NA	
Tsougos I, et al. ^38^ 2012	Differentiation of glioblastoma multiforme from metastatic brain tumor using proton magnetic resonance spectroscopy, diffusion and perfusion metrics at 3 T	Intratumoral	11.49 ± 6.33	10.80 ± 5.13	0.147 ± 0.065	0.119 ± 0.047	49
		Peritumoral	1.68 ± 0.59	1.06 ± 0.38	0.291 ± 0.075	0.261 ± 0.063	
Tsuchiya K, et al. ^39^ 2005	Differentiation between solitary brain metastasis and high-grade glioma by diffusion tensor imaging	Intratumoral	NA	NA	0.16 ± 0.05	0.14 ± 0.05	14
		Peritumoral	NA	NA	0.20 ± 0.09	0.16 ± 0.05	
Lu S, et al. ^40^ 2004	Diffusion-tensor MR imaging of intracranial neoplasia and associated peritumoral edema: introduction of the tumor infiltration index	Intratumoral	NA	NA	0.205 ± 0.043	0.226 ± 0.092	20
		Peritumoral	NA	NA	0.243 ± 0.043	0.211 ± 0.033	
Law M, et al. ^16^ 2002	High-grade gliomas and solitary metastases: differentiation by using perfusion and proton spectroscopic MR imaging	Intratumoral	2.87 ± 1.89	3.05 ± 1.79	NA	NA	36
		Peritumoral	1.31 ± 0.97	0.39 ± 0.19	NA	NA	
Bulakbasi N., et al. ^41^ 2005	Assessment of diagnostic accuracy of perfusion MR imaging in primary and metastatic solitary malignant brain tumors	Intratumoral	5.42 ± 1.52	3.21 ± 0.98	NA	NA	39
		Peritumoral	2.17 ± 0.82	0.97 ± 0.09	NA	NA	
Shi, L et al. ^42^ 2010	Diffusion tensor MRI in ring-enhancing cerebral lesions	Intratumoral	NA	NA	0.069 ± 0.02	0.064 ± 0.02	43
		Peritumoral	NA	NA	0.236 ± 0.06	0.171 ± 0.06	

DSC, dynamic susceptibility contrast; DWI, diffusion-weighted imaging; rCBV, relative cerebral blood volume.

**Table 2.** Extracted data of DSC metrics rCBV variable in the intratumoral and peritumoral regions in included studies

Study Author (Study year)	Title	Area peritumoral/ intratumoral	High-grade glioma rCBV (mean ± SD)	No. of participants	Solitary brain metastasis rCBV ((mean ± SD)	No. of participants	Total participants
She D, et al. ^27^ 2019	Differentiation of glioblastoma and solitary brain metastasis by gradient of rCBV in the peritumoral brain zone derived from DSC perfusion MRI	Intratumoral	NA	24	NA	19	43
		Peritumoral	0.73 ± 0.37		0.51 ± 0.24		
Mouthuy N, et al. ^28^ 2012	Multiparametric MRI to differentiate high-grade gliomas and brain metastases	Intratumoral	10.7 ± 5.3	38	6.63 ± 4.61	8	46
		Peritumoral	1.91 ± 1.69		0.74 ± 0.49		
Svolos P, et al. ^30^ 2013	Investigating brain tumor differentiation with diffusion and perfusion metrics at 3T MRI using pattern recognition techniques	Intratumoral	7.14 ± 2.33	53	7.80 ± 2.61	18	71
		Peritumoral	2.67 ± 1.06		0.94 ± 0.35		
Blasel S, et al. ^7^ 2010	Elevated peritumoural rCBV values as a mean to differentiate metastases from high-grade gliomas	Intratumoral	NA	29	NA	23	52
		Peritumoral	1.17 ± 0.32		0.78 ± 0.17		
Aslan K, et al. ^31^ 2019	Multiparametric MRI in differentiating solitary brain metastasis from high-grade glioma: diagnostic value of the combined use of DWI, DSC imaging, and magnetic resonance spectroscopy parameters	Intratumoral	3.63 ± 1.40	39	3.68 ± 1.40	17	56
		Peritumoral	1.14 ± 0.46		0.39 ± 0.18		
Tsolaki E, et al. ^25^ 2013	Automated differentiation of glioblastomas from intracranial metastases using 3T MR spectroscopic and perfusion data	Intratumoral	7.13 ± 3.17	35	7.73 ± 4.36	14	49
		Peritumoral	2.81 ± 1.44		1.29 ± 0.61		
Bauer AH, et al. ^2^ 2015	Differentiation of solitary brain metastasis from glioblastoma multiforme: a predictive multiparametric approach using combined MR diffusion and perfusion	Intratumoral	3.87 ± 1.17	13	2.55 ± 1.20	10	23
		Peritumoral	1.71 ± 1.21		0.94 ± 1.25		
Neska-Matuszewska M, et al. ^33^ 2018	Differentiation of glioblastoma multiforme, metastases and primary central nervous system lymphomas using multiparametric perfusion and diffusion MR imaging of a tumor core and a peritumoral zone-Searching for a practical approach	Intratumoral	3.10 ± 1.50	27	4.49 ± 3.94	30	57
		Peritumoral	1.05 ± 0.39		0.55 ± 0.13		
Chiang IC, et al. ^15^ 2004	Distinction between high-grade gliomas and solitary metastases using peritumoral 3 T magnetic resonance spectroscopy, diffusion, and perfusion imagings	Intratumoral	0.09 ± 0.05	14	0.22 ± 0.23	12	26
		Peritumoral	2.33 ± 1.61		0.84 ± 0.33		
Zhang H., et al. ^34^ 2009	Differentiation between supratentorial single brain metastases and high grade astrocytic tumors: an evaluation of different DSC MRI measurements	Intratumoral	6.00 ± 2.17	24	2.75 ± 1.72	29	53
		Peritumoral	1.77 ± 1.19		1.05 ± 0.53		
Svolos P., et al. ^35^ 2013	Classification methods for the differentiation of atypical meningiomas using diffusion and perfusion techniques at 3 T MRI	Intratumoral	10.95 ± 6.55	15	8.92 ± 3.61	27	42
		Peritumoral	1.81 ± 0.59		1.23 ± 0.38		
Cindil E., et al. ^37^ 2021	Validation of combined use of DWI and percentage signal recovery-optimized protocol of DSC-MRI in differentiation of high-grade glioma, metastasis, and lymphoma	Intratumoral	4.01 ± 2.51	60	4.25 ± 3.05	24	84
		Peritumoral	1.61 ± 0.99		0.77 ± 0.31		
Tsougos I, et al. ^38^ 2012	Differentiation of glioblastoma multiforme from metastatic brain tumor using proton magnetic resonance spectroscopy, diffusion and perfusion metrics at 3 T	Intratumoral	11.49 ± 6.33	35	10.80 ± 5.13	14	49
		Peritumoral	1.68 ± 0.59		1.06 ± 0.38		
Law M, et al. ^16^ 2002	High-grade gliomas and solitary metastases: differentiation by using perfusion and proton spectroscopic MR imaging	Intratumoral	2.87 ± 1.89	24	3.05 ± 1.79	12	36
		Peritumoral	1.31 ± 0.97		0.39 ± 0.19		
Bulakbasi N., et al. ^41^ 2005	Assessment of diagnostic accuracy of perfusion MR imaging in primary and metastatic solitary malignant brain tumors	Intratumoral	5.42 ± 1.52	22	3.21 ± 0.98	17	39
		Peritumoral	2.17 ± 0.82		0.97 ± 0.09		

DSC, dynamic susceptibility contrast; DWI, diffusion-weighted imaging; rCBV, Relative cerebral blood volume.

**Table 3.** Extracted data of DWI metric FA variable in the intratumoral and peritumoral regions in included studies

Study Author (Study year)	Title	Area peritumoral/ intratumoral	High-grade glioma FA (mean ± SD)	No. of participants	Solitary brain metastasis FA (mean ± SD)	No. of participants	Total participants
Mao J, et al.^9^ 2020	Differentiation between high-grade gliomas and solitary brain metastases: a comparison of five diffusion-weighted MRI models	Intratumoral	0.33 ± 0.13	20	0.22 ± 0.09	21	41
		Peritumoral	0.35 ± 0.11		0.33 ± 0.15		
Kadota Y, et al. ^26^ 2020	Differentiation between glioblastoma and solitary brain metastasis using neurite orientation dispersion and density imaging	Intratumoral	0.145 ± 0.082	9	0.098 ± 0.042	6	15
		Peritumoral	0.170 ± 0.024		0.158 ± 0.065		
Abdel Razek AAK, et al. ^29^ 2019	Differentiating glioblastomas from solitary brain metastases using arterial spin labeling perfusion- and diffusion tensor imaging-derived metrics	Intratumoral	0.14 ± 0.04	21	0.17 ± 0.03	15	36
		Peritumoral	0.22 ± 0.08		0.32 ± 0.07		
Svolos P, et al. ^30^ 2013	Investigating brain tumor differentiation with diffusion and perfusion metrics at 3T MRI using pattern recognition techniques	Intratumoral	0.148 ± 0.058	53	0.117 ± 0.040	18	71
		Peritumoral	0.286 ± 0.069		0.251 ± 0.048		
Tan Y, et al. ^32^ 2015	Differentiation of high-grade astrocytomas from solitary brain metastases: Comparing diffusion kurtosis imaging and diffusion tensor imaging	Intratumoral	0.21 ± 0.20	31	0.18 ± 0.07	20	51
		Peritumoral	0.18 ± 0.05		0.16 ± 0.03		
Bauer AH, et al. ^2^ 2015	Differentiation of solitary brain metastasis from glioblastoma multiforme: a predictive multiparametric approach using combined MR diffusion and perfusion	Intratumoral	0.23 ± 0.04	13	0.11 ± 0.05	10	23
		Peritumoral	0.27 ± 0.05		0.16 ± 0.05		
Svolos P., et al. ^35^ 2013	Classification methods for the differentiation of atypical meningiomas using diffusion and perfusion techniques at 3 T MRI	Intratumoral	0.140 ± 0.052	15	0.116 ± 0.040	27	42
		Peritumoral	0.291 ± 0.085		0.279 ± 0.046		
Lu S., et al. ^36^ 2003	Peritumoral diffusion tensor imaging of high-grade gliomas and metastatic brain tumors	Intratumoral	NA	12	NA	12	24
		Peritumoral	0.248 ± 0.063		0.181 ± 0.041		
Tsougos I, et al. ^38^ 2012	Differentiation of glioblastoma multiforme from metastatic brain tumor using proton magnetic resonance spectroscopy, diffusion and perfusion metrics at 3 T	Intratumoral	0.147 ± 0.065	35	0.119 ± 0.047	14	49
		Peritumoral	0.291 ± 0.075		0.261 ± 0.063		
Tsuchiya K, et al. ^39^ 2005	Differentiation between solitary brain metastasis and high-grade glioma by diffusion tensor imaging	Intratumoral	0.16 ± 0.05	7	0.14 ± 0.05	7	14
		Peritumoral	0.20 ± 0.09		0.16 ± 0.05		
Lu S, et al. ^40^ 2004	Diffusion-tensor MR imaging of intracranial neoplasia and associated peritumoral edema: introduction of the tumor infiltration index	Intratumoral	0.205 ± 0.043	10	0.226 ± 0.092	10	20
		Peritumoral	0.243 ± 0.043		0.211 ± 0.033		
Shi, L et al. ^42^ 2010	Diffusion tensor magnetic resonance imaging in ring-enhancing cerebral lesions	Intratumoral	0.069 ± 0.02	35	0.064 ± 0.02	8	43
		Peritumoral	0.236 ± 0.06		0.171 ± 0.06		

DWI, diffusion-weighted imaging; FA, Fractional anisotropy.

### Risk of bias assessment

Newcastle-Ottawa quality assessment Scale (NOS) and Cochrane risk of bias tool (ROB) for observational and clinical trial studies, were managed to evaluate the quality of the research paper included. (Table 4 **NOS for assessing the quality of observational studies**, and Table 5 **ROB for quality appraisal the clinical studies**)

**Table 4.** . Newcastle-Ottawa scale for assessing the quality of observational studies

Studies ID	Selection	Comparability	Outcome	Overal
She D, et al. 2019^27^	3	2	3	Good
Mouthuy N, et al. 2012^28^	4	2	3	Good
Aslan K, et al., 2019^31^	3	1	3	Good
Tan Y, 2015^32^	4	2	3	Good
Bauer AH,et al., 2015^2^	4	2	3	Good
Neska-Matuszewska M,et al., 2018^33^	3	2	3	Good
Zhang H., et al., 2009^34^	3	1	3	Good
Lu S., etal., 2003^36^	4	1	3	Good
Cindil E., et al., 2021^37^	4	2	3	Good
Tsuchiya K, et al. 2005^39^	3	2	3	Good
Lu S, et al. 2004^40^	4	2	3	Good

**Table 5.** Risk of bias tool for quality appraisal the clinical studies

Studies ID	Selection bias	Performance bias	Detection bias	Attrition bias	Reporting bias	Other bias	Overal risk of bias
Mao J, et al., 2020^9^	Low risk	Low risk	Low risk	Low risk	Low risk	Low risk	Low risk
Kadota Y, et al., 2020^26^	Low risk	Low risk	Low risk	Low risk	Low risk	Low risk	Low risk
Abdel Razek AAK, et al., 2019^29^	Low risk	Low risk	Low risk	Low risk	Low risk	Low risk	Low risk
Svolos P, et al., 2013^30^	Low risk	Low risk	Low risk	Low risk	Low risk	Low risk	Low risk
Blasel S, Jet al., 2010^7^	Low risk	Low risk	Low risk	Low risk	Low risk	Low risk	Low risk
Tsolaki E, et al., 2013^25^	Low risk	Low risk	Low risk	Low risk	Low risk	Low risk	Low risk
Chiang IC,et al., 2004^15^	Low risk	Low risk	Low risk	Low risk	Low risk	Low risk	Low risk
Svolos P., et al., 2013^30^	Low risk	Low risk	Low risk	Low risk	Low risk	Low risk	Low risk
Tsougos, I., et al., 2012^38^	Low risk	Low risk	Low risk	Low risk	Low risk	Low risk	Low risk
Law M, et al^16^.	Low risk	Low risk	Low risk	Low risk	Low risk	Low risk	Low risk
Bulakbasi N., et al. 2005^41^	Low risk	Low risk	Low risk	Low risk	Low risk	Low risk	Low risk
Shi, L et al. 2010^42^	Low risk	Low risk	Low risk	Low risk	Low risk	Low risk	Low risk

The risk of bias was evaluated methodologically by two reviewers, and conflicts were resolved through discussion.

## Strategy for data synthesis

### Statistical analysis

Summary tables of characteristics and outcome data from the included studies provided a qualitative overview. Pooling studies were synthesized quantitatively into the meta-analysis. The research paper that fulfilled full-text inclusion criteria but did not have mean and standard deviation were excluded. Besides, subgroup analysis was conducted discriminating HGG and SBM using perfusion MR to measure rCBV, and diffusion MR measures FA in intratumoral and peritumoral regions. Heterogeneity data were included qualitatively but not synthesized quantitatively.

**Figure 1.**
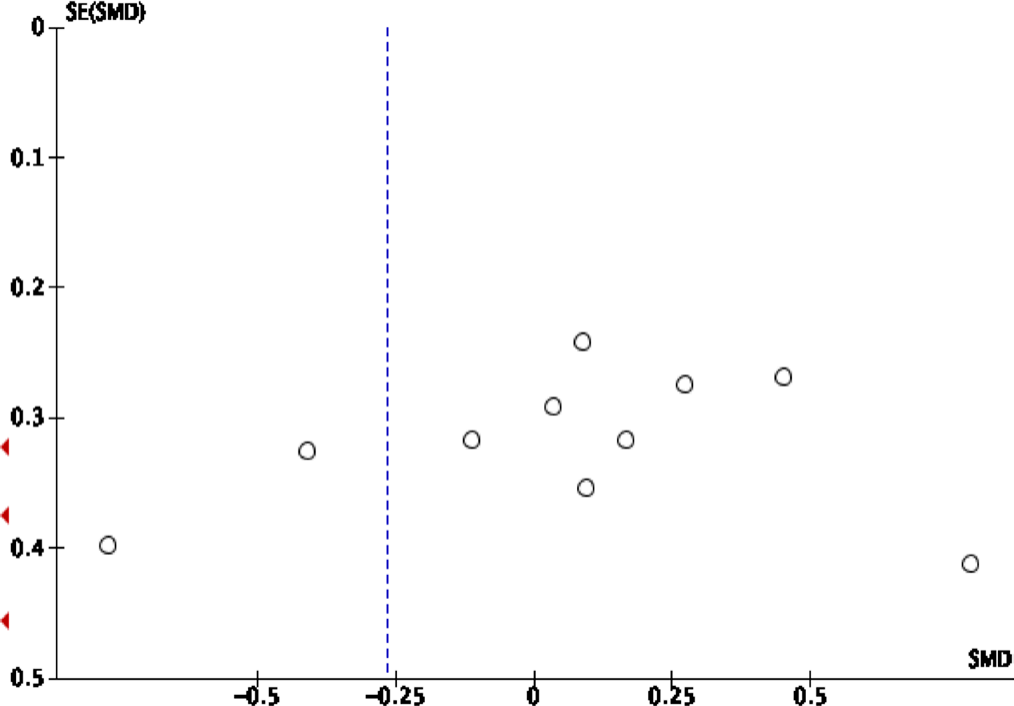
Funnel plot standard mean difference of rCBV intratumoral HGG *vs* MET. HGG, high-grade glioma; rCBV, relative cerebral blood volume.

**Figure 2.**
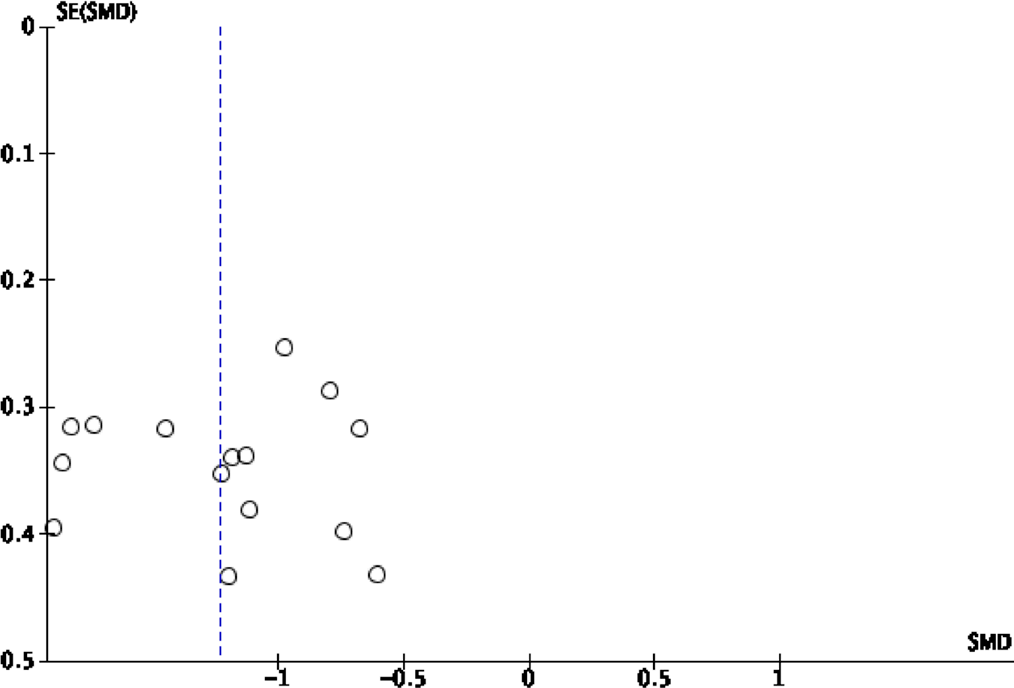
Funnel plot standard mean difference of rCBV in peritumoral HGG *vs* MET. HGG, high-grade glioma; rCBV, relative cerebral blood volume.

**Figure 3.**
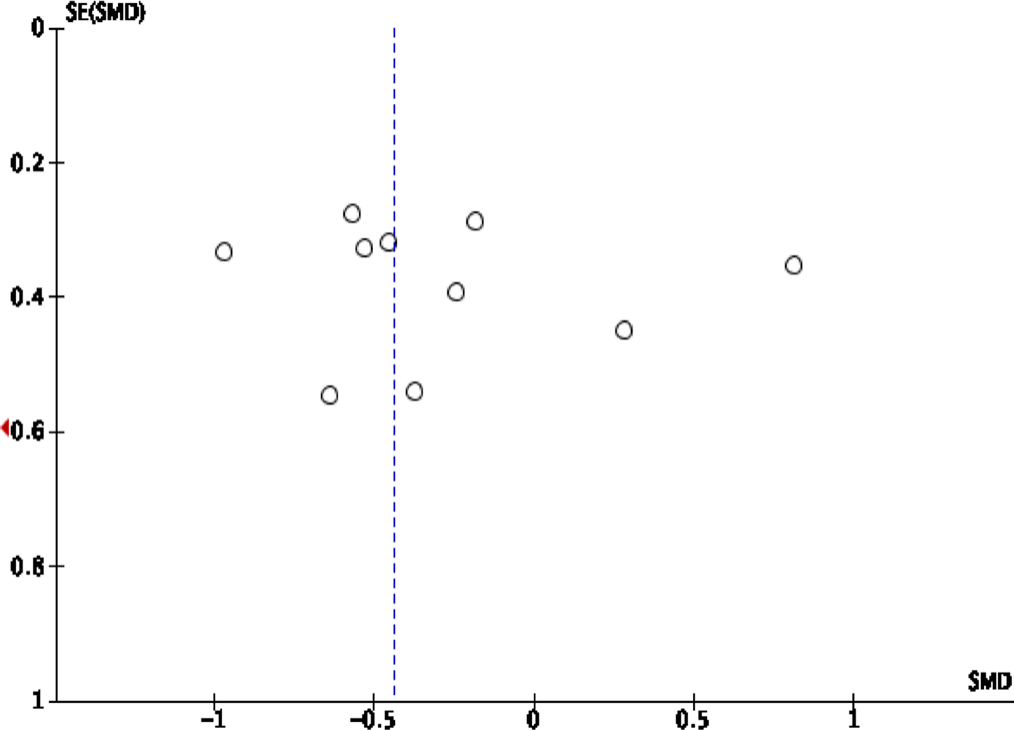
Funnel plot standard mean difference of FA in intratumoral HGG *vs* MET. FA, fractional anisotropy; HGG, high-grade glioma.

**Figure 4.**
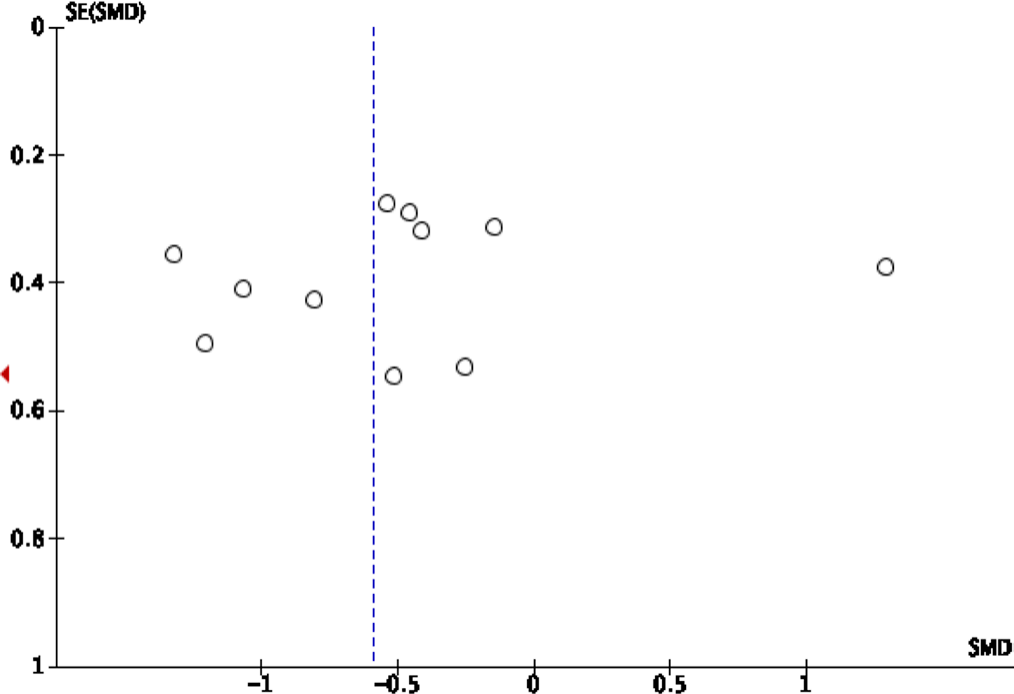
Funnel plot standard mean difference of FA in peritumoral HGG *vs* MET. FA, fractional anisotropy; HGG, high-grade glioma.

Review Manager 5.4^43^ was used in this study to manage data synthesis quantitatively for studies on conditions of interest. Random effects meta-analyses, standard mean difference, and their 95% CI for the different parameters analyzed from individual studies were used to assess the discrimination between HGG and SBM. Heterogeneity was evaluated using the Higgins I2 statistic, which measures the percentage of the total variation across the included studies.^44^ The values of I2 lie between 0 and 100%. A value of 0% indicates no heterogeneity. We divided heterogeneity is no heterogeneity (I2 <25%), low (25 ≤ I2 <50%), moderate (50 ≤ I2 <75%), and high (I2 ≥75%) (166, 167). A funnel plot was used to examine the publication bias visually (Figure 1, Figure 2, Figure 3, Figure 4).

## Results

### Search results

**Figure 5.**
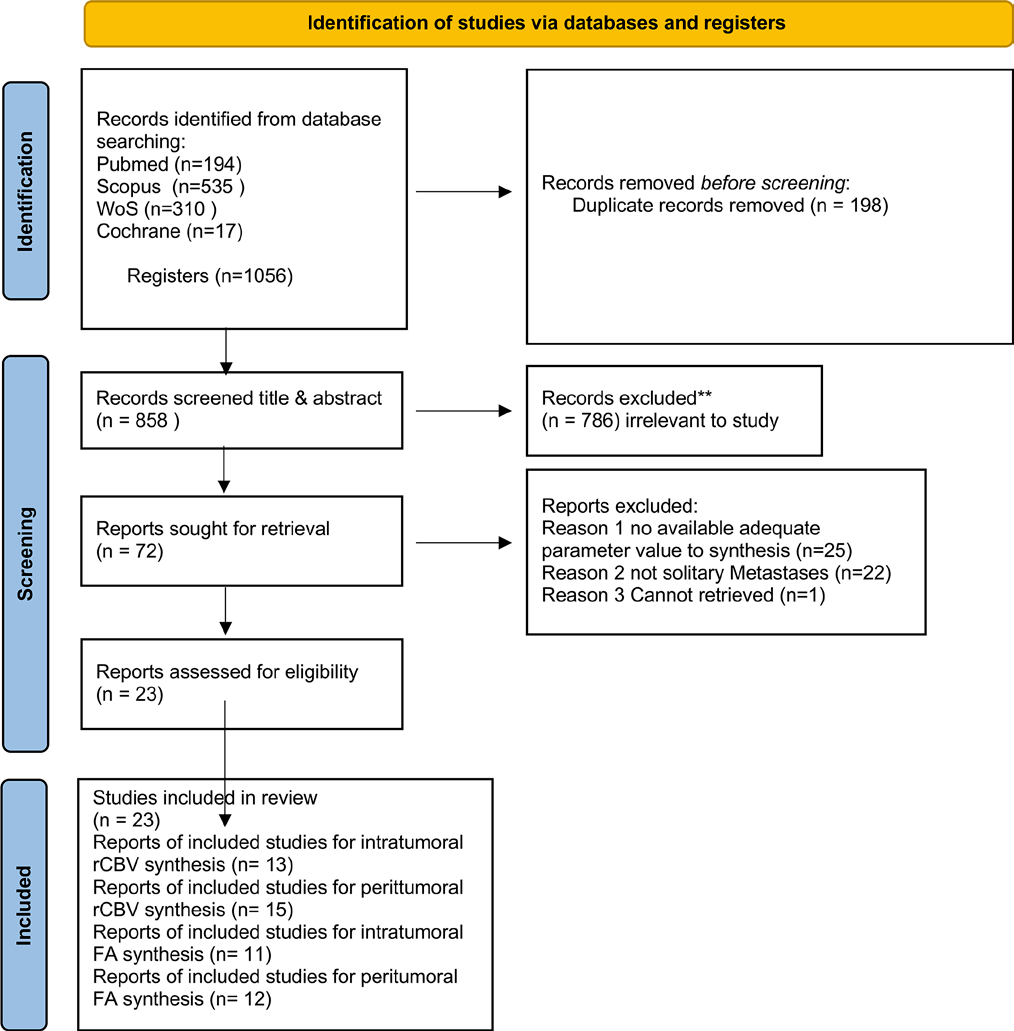
Prisma flow diagram differentiation of high-grade glioma and solitary brain metastases measure of rCBV & FA. FA, fractional anisotropy; rCBV, relative cerebral blood volume.

There are 1056 studies in total found by searching the databases after inclusion and exclusion criteria filtered by automation tools (*n* = 218); 194 were acquired from PubMed, 535 from Scopus, 310 from Web of Science, and 17 from Cochrane library. One hundred ninety-eight (198) duplicates were removed using Mendeley and Excel, and screened manually for similar research paper titles. After duplicate removal, 858 studies were screened for the title and abstract relevancy, and 786 were excluded due to irrelevant to our study, as most of them did not exactly report the difference between HGG and SBM using rCBV and FA value. From the above screening, 72 studies were included for full-text evaluation, of which 49 were excluded, and 23 studies were enrolled for qualitative and quantitative data synthesis. A Summary of search results is shown in Figure 5.

### Characters of the included studies

The qualitative and quantitative data synthesis enrolled 23 studies in total^2,7,9,15,16,25–42^ done from 2002^45^ to 2020^9^ 12 studies were prospectively conducted^7,9,15,16,25,26,29,30,35,38,41,42^ and 11 studies were retrospective in nature,^2,27,28,31–34,36,37,39,40^ with a total of 970 participants. The characteristics of the populations were patients with HGG (*n* = 597) and patients with SBM (*n* = 373), whereas the remaining data were based on MRI measuring of perfusion MR variable rCBV and diffusion MR variable FA. Based on the risk of bias evaluation, all of the enrolled studies are of good quality for analysis.

**Table 6.** Summarizes the characters of the included studies

Study Author (Study year)	Study design	Population type	No. of participants	Mean age (years)	Age range	High-grade glioma (n))	Solitary brain metastasis (n)	Country	Field Strength	Area peritumoral/ intratumoral	MRI Measurement / variable	Condition	Outcome
Mao J, et al.^9^ 2020	Prospective study	Brain tumors [Solitary High-grade glioma, SBM	41	HGG (55.70), SBM (54.05)	HGG (19-67), SBM (43.81)	20	21	China	3.0 T	Contrast Enhancing Tumor & Peritumoral oedem	NODDI, MAP-MRI, DKI, DTI and DWI.	HGGs [7 AA (WHO grade III) and 13 GB (WHO grade IV)]. SBMs [10 lung carcinoma, five breast carcinoma, three colon carcinoma, one liver carcinoma, one gastric carcinoma, and one thyroid carcinoma].	For NODDI, MAP-MRI, DKI, and DTI, the best single discriminative parameters were isotropic volume fraction (Viso), mean-squared displacement (MSD), Diffusion Kurtosis Imaging (DKI)-generated radial (RDk), and DTI-generated radial (RD), respectively. Viso had a substantially higher AUC (0.871) than MSD (0.736), RDk (0.760), and RD (0.733) (*p* < 0.05).
Kadota Y, et al. ^26^ 2020	Prospective study	Brain tumors [GB, SBM]	15	GB (66.1), SBM (55.7)	GB (44-79), SBM (38-79)	9	6	Japan	3.0 T	peritumoral signal-change (PSC) – and the enhancing solid area of the lesion.	NODDI intra cellular, extra cellular, and isotropic volume (VIC, VEC, VISO) fraction. Diffusion data (ADC, FA)	six brain metastases, the primary tumors were [5non-small-cell lung carcinomas, the other patient the primary site was unknown.]	The mean value of the PSC area on VEC maps was substantially larger for glioblastoma than metastasis (*p* < 0.05), whereas on VISO maps it tended to be higher for metastasis than glioblastoma. On the other maps, there was no discernible change. The VEC fraction in the PSC region had the best diagnostic performance of the five measures. For distinguishing between the two tumor types, the VEC threshold value of 0.48 gave 100% sensitivity, 83.3% specificity, and an AUC of 0.87.
She D, et al. ^27^ 2019	Retrospective study	Brain tumors [GB, SBM]	43	NA	NA	24	19	China	3.0 T	enhancing tumoral, & peritumoral area, near the enhancing tumor, G1; intermediate distance from the enhancing tumor, G2; far from the enhancing tumor, G3	DSC-MRI (rCBV) ratio data in three regions	SBMs s [15 lung carcinomas, one renal carcinoma, one gastric carcinoma, one intes- tinal carcinoma, and one melanoma.]	GB had substantially greater rCBVp ratios and rCBV gradient in the Peritumoral brain zone (PBZ) than SBM (*p* < 0.05 for both rCBVp ratios and rCBV gradient). rCBVp ratios with threshold values of 0.50 or above had sensitivity and specificity of 57.69 and 79.17%, respectively, for distinguishing GB from SBM. Using a threshold value of larger than 0.06, the rCBV gradient exhibited better sensitivity (94.44%) and specificity (91.67%) than rCBVp ratios.
Mouthuy N, et al. ^28^ 2012	Retrospective study	Brain tumors [GBM, SBM]	46	Median age 60 years	29–84	38	8	Belgium	1.5 T & 3.0 T	Enhancing ring like tumoral & peritumoral	PWI, (T2/FLAIR/T1) perfusion (rCBV),	38 of the lesions were HGGs [11 high-grade astrocytomas, and 27 GB] 9 of them multifocal, (8 SBMs. 6 were singles, 2 of which were infratentorial.	Between SBM and GBM, there were significant statistical differences in circularity, surface area, rCBVs, percentage of signal intensity recovery, and texture characteristics (energy, entropy, homogeneity, correlation, inverse differential moment, sum average) (*p* < 0.05). With these settings, we were able to achieve moderate-to-good categorization results. With a sensitivity of 92% and a specificity of 71 %, clustering based on rCBV and textural characteristics (contrast, sum average) distinguished SBM from GBM.
Abdel Razek AAK, et al. ^29^ 2019	Prospective study	Brain tumors [GBM, SBM]	36	NA	NA	21	15	Egypt	1.5 T	Enhancing tumoral & peritumoral	TBF & DTI (FA, MD)	15 brain metastasis [seven breast cancer, four bronchogenic carcinoma, three gastrointestinal tumors, and one thyroid cancer.	TBF (*p* = 0.001) and MD (*p* = 0.001) of the tumoral and peritumoral portions of glioblastoma, as well as metastasis (*p* = 0.001), were significantly different. Between glioblastomas and metastasis, there was a significant difference in FA of the peritumoral portion (*p* = 0.001) but an insignificant difference in FA of the tumoral part (*p* = 0.06). TBF cutoffs for tumoral and peritumoral portions utilized for differentiation were 29.7 and 17.8 (mL/100 g/minute), respectively, yielding AUCs of 0.943 and 0.937, respectively, with 91.7 and 88.9% accuracy. The MD cutoffs for tumoral and peritumoral portions were 1.27 and 1.33 (10^3^mm^2^/second), respectively, revealing AUCs of 0.840 and 0.987 and accuracy of 83.3% and 91.7%. The peritumoral part’s combined TBF, MD, and FA indicated an AUC of 0.984 and accuracy of 91.7 percent.
Svolos P, et al. ^30^ 2013	prospective clinical study	Brain tumors [LGG, HGG, SBM, MenIingioma]	71	NA	31–77	53	18	Greece	3.0 T	intratumoral & peritumoral	Diffusion:DWI parameter, DTI parameter and Perfussion: DSCI parameter	53 HGGs (12 Grade III, 41 Grade IV), eight metastatic lesions [ 13 lung, and five breast primary tumors.]	The Support Vector Machine (SVM) classification produced the best predicted results, while Receiver operating characteristic (ROC) analysis also produced excellent accuracies. DWI/DTI and DSCI are clearly helpful methods for tumor grading. Nonetheless, cellularity and vascularity are non-linearly linked variables that are challenging to assess and understand using traditional techniques of research. As a result, combining diffusion and perfusion measurements into a complex classification method may yield the best diagnostic result.
Blasel S, et al. ^7^ 2010	Prospective study (clinical)	Brain tumors [GB, SBM]	52	GB (58.7), SBM (65.3)	GB (23–29), SBM (41–75)	29	23	Germany	3.0 T	Peritumoral & contralateral normal white matter	rCBV values	29 Solitary GB, 23 metastasis [ 10 lung, two breast, two colon, four melanoma, one prostate, one chondrosarcoma, one gastric, one ovary, and one unknown primary.]	In metastases, peritumoural rCBV was considerably lower than in GB (*p* < 0.01). The cutoff value of 1.0 had a sensitivity of 96 %, a specificity of 64 %, a positive predictive value of 68 %, and a negative predictive value of 95% for distinguishing metastases from GB.
Aslan K, et al. ^31^ 2019	Retrospective study	Brain tumors [HGG, SBM]	56	HGG (61.2 ± 10.5 years); SBM (61.0 ± 13.8 years)	HGG (37–81 yearss), SBM (29–83 year)	39	17	Turkey	1.5 T	enhencing tumor & peritumoral edema	DWI (ADCmin, ADCmax, and ADCmean), DSCI (rCBV), and MRS (Cho/Cr, Cho/NAA, and NAA/Cr)	39 HGG [11 with WHO grade III (8 AA and three anaplastic oligodendroglioma) and 28 with WHO grade IV (glioblastomas). 17 Metastatic brain tumours [nine lung carcinoma, three breast carcinoma, one melanoma, one renal carcinoma, one colon carcinoma, one ovarian carcinoma, and 1 carcinoma of unknown origin.]	All of the measures in the enhancing tumor, with the exception of NAA/Cr (*p* = 0.024), showed no significant difference in separating these two groups (*p* > 0.05). In the peritumoural area, AUC values for ADCmin, ADCmax, ADCmean, rADCmin, rADCmax, rADCmean, rCBV, Cho/Cr, Cho/NAA, and NAA/Cr parameters in distinguishing SBM from HGG were 0.860, 0.822, 0.848, 0.822, 0.801, 0.822, 0.906, 0.851, 0.903, The best model for distinguishing HGG from SBM was a mix of peritumoural ADCmin, rCBV, and Cho/NAA factors. The AUC value was 0.970.
Tan Y, et al. ^32^ 2015	Retrospective study	Brain tumors [HG Astrocytoma, SBM]	51	HGA (56.6 ± 12.5), SBM (60.1 ± 13.4)	HGA (39 to 70 years), SBM (40 to 77 years)	31	20	China	3.0 T	tumoral, peritumoral & contra lateral Normal white Matter (NAWM)	DKI (MK, Kr, and Ka) and DTI (FA and MD)	20 brain metastases, the primary sites were [12 the lung, four breast, one thyroid, one kidney, and two colon.]	There were no significant variations in DKI values (MK, Kr, and Ka) or DTI values (FA and MD) in tumoral solid portions between the two groups. High-grade astrocytomas had substantially greater corrected and uncorrected MK, Kr, and Ka values in peritumoral edema than solitary-brain-metastases, and MD values without adjustment were lower in high-grade astrocytomas than solitary-brain-metastases. Corrected Ka (1.000), MK (0.889), and Kr (0.880) values had substantially larger areas under curve (AUC) than MD (0.793) and FA (0.472) values. For adjusted MK, Kr, Ka, and MD, the optimum thresholds were 0.369, 0.405, 0.483, and 2.067, respectively.
Tsolaki E, et al. ^25^ 2013	Prospective clinical study	Brain tumors [GBM, SBM]	49	NA	32–73 years	35	14	Greece	3.0 T	intratumoral & peritumoral	Metabolic (NAA/Cr, Cho/Cr, (Lip ++Lac)/Cr) and perfusion (rCBV)	14 metastatic lesions [12 lung, and two breast primary tumors.]	Only in the peritumoral area of these lesions were glioblastoma and metastases distinguishable (*p* < 0.05). For both the intratumoral and peritumoral regions, SVM had the best overall performance (accuracy 98%). The performance of Nave-Bayes and KNN was more variable. Because datasets are intimately connected to the underlying pathophysiology, effective dataset selection is critical.
Bauer AH, et al. ^2^ 2015	Retrospective study	Brain tumors [GBM, SBM]	23	NA	32–78 years	13	10	USA	3.0 T	enhancing tumoral & non-enhancing peritumoral	DTI, DCE, and DSC perfusion (FA,MD), *K* trans, and rCBV	10 SBMs [four non-small cell lung adenocarcinoma, one colon adenocarcinoma, two breast adenocarcinoma, one melanoma, one ovarian serous adenocarcinoma, and one neuroendocrine tumor].	In GBM, rCBV, K trans, and FA were greater in the augmenting tumor, but MD was decreased, both without statistical significance. In the non-enhancing peritumoral T2 hyperintense region (NET2), GBM had considerably greater rCBV (*p* = 0.05), but significantly lower MD (*p* < 0.01). FA and K trans levels were greater in GBM, although not statistically significant. In NET2, a combination of rCBV, FA, and MD produced the greatest discriminative power, with an area under the curve (AUC) of 0.98.
Neska-Matuszewska M, et al. ^33^ 2018	Retrospective cohort	Brain tumors [GBM, SBM, PCNSL]	57	GBM (61 years)), SBM (64.5years)	NA	27	30	UK	1.5 T	tumoral core & peritumoral	rCBV, rPH, rPSR and ADC	16 metastases from lung cancer, four from renal cancer, two from intestinal cancer, five from breast cancer and 3 were of an unknown origin.	There were no changes in perfusion and diffusion characteristics between GBMs and metastases inside the tumor core. PCNSLs had considerably lower rCBV and peak height (rPH), ADC, and higher percentage of signal recovery (rPSR) values than GBMs and metastases. Max rCBV had the greatest accuracy of 0.98 in distinguishing PCNSLs from other tumors, with a cut-off value of 2.18. The peritumoral zone was analyzed to identify GBMs from metastases, with substantially greater rCBV, rPH, and lower ADC values in GBMs, with the best accuracy of 0.94 reported for max rCBV at a cut-off value of 0.98.
Chiang IC, et al. ^15^ 2004	Prospective	Brain tumors [HGG/GBM, SBM]	26	NA	25–76 years	14	12	Taiwan	3.0 T	tumoral & peritumoral	MRS, diffusion imaging, and conventional MR imaging. ADC values, rCBV values. Cho/Cr, NAA/Cr,	14 HGGs [ GBMs]. 12 SBMs all were carcinomas [nine known primary (two breast, five lung, two stomach), and three from an unknown primary site.]	In the peritumoral areas of high-grade gliomas, the choline to creatine ratio and relative cerebral blood volume were substantially greater than in the metastases. The apparent diffusion coefficient values in metastasis tumoral and peritumoral areas were considerably greater than in original gliomas. Although the features of isolated metastases and original high-grade gliomas on conventional MR imaging can be confusing at times, peritumoral perfusion-weighted and spectroscopic MR imaging can help distinguish the two.
Zhang H., et al. ^34^ 2009	Retrospective study	Brain tumors [Gliomas, SBM]	53	49	24–72	24	29	China	1.5 T	tumoral & peritumoral oedema	DSC MRI [CBF (T rCBV, T rCBF and T rMTT) and (P rCBV, P rCBF and P rMTT)	24 HGG (17 GBs, 7 AA). 29 metastatic tumors (15 lung, eight breast, 4 gastric and two renal cancer).	Tumoral relative Cerebral Blood Volume (T rCBV), T rCBF, P rCBV, and Peritumoral cerebral blood flow (P CBF) of brain metastases (2.75 ± 1.72, 2.51 ± 2.09, 1.05 ± 0.53, 0.87 ± 0.40) were statistically different (*p* < 0.05) from those of high grade astrocytic tumors (6.00 ± 2.17, 5.68 ± 2.35, 1.77 ± 1.19, and 1.58 ± 0.99). There was no significant difference between these two entities' mean rMTTs (*p* > 0.05). The efficiency of T rCBV and T rCBF for accurate diagnosis of brain metastases is virtually equal (AUC: 0.899, 0.890, respectively) and superior to other measures, according to the area under the ROC curves (AUC). T rCBF had the same specificity (86.7) as T rCBV, but better sensitivity (86.2) and accuracy (86.2) with a threshold value of 3.50. (86.3). Single metastases and high-grade astrocytic tumors can be distinguished using various perfusion measures.
Svolos P., et al. ^35^ 2013	prospective clinical study	Brain tumors [Meningioma, GB, SBM ]	42	NA	20–77	15	27	Greece	3.0 T	intratumoral & peritumoral	ADC, FA and rCBV	Solitary Glioblastomas, and Solitary metastases	The use of categorization algorithms increases the usefulness of differential diagnostics incrementally. Diffusion measures are mostly used for differentiation, however perfusion measurements may give useful information for the peritumoral areas.
Lu S., et al. ^36^ 2003	Retrospective study	Brain tumors [GBM, SBM]	24	50.0 years ± 14.2	28–77 years	12	12	USA	1.5 T	peritumoral & normal appearing white matter	DTI (FA & MD)	12 HGGs [9 GBM, and 3 AA], 12 metastatic brain lesions [ five lung carcinomas, two breast carcinomas, two melanomas, one testicular yolk sac tumor, one osteogenic sarcoma, and one undifferentiated sarcoma.]	When gliomas and metastatic tumors were compared to normal-appearing white matter, the peritumoral area showed substantial increases in MD (*p* < .005) and significant decreases in FA (*p* < .005). Furthermore, metastatic lesions' peritumoral MD was considerably higher than that of gliomas (*p* < .005). Measurements of peritumoral FA, on the other hand, revealed no such difference.
Cindil E., et al. ^37^ 2021	Retrospective study	Brain tumors [PCNSL, HGG, SBM]	84	HGGs (50 years ± 16), 24 metastases (57 years ± 12), and 15 PCNSLs (61 years ± 15)	NA	60	24	Turkey	3.0 T	Tumoral core & peritumoral oedema	DSC-MRI, PSR- and DWI. (rCBV, PSR, ADC.)	Solitary HGGs [40 GBs, and 20 AA], 12 Solitary metastases [12 lung cancer, seven breast cancer, two rectal cancer, one thyroid cancer, one gastric adenocarcinoma, and one from malign melanoma].	With AUC values of 0.979 for PCNSL *vs* others and 0.947 for HGG *vs* metastases, PSR in the tumor core had the greatest discriminating performance in differentiating these three tumor types. The ADC was only useful for differentiating PCNSLs from other PCNSLs in the tumor core (AUC = 0.897).
Tsougos I, et al. ^38^ 2012	Prospective cliniccal study	Brain tumors [GB, SBM]	49	NA	32–73	35	14	Greece	3.0 T	Intratumoral, peritumoral, contra lateral normal area	N-acetylaspartate (NAA)/creatine (Cr), choline (Cho)/Cr, Cho/NAA, rCBV, ADC and FA	Solitary glioblastoma, solitary metastases consisted of [6 lung and eight breast primary tumors.]	Glioblastomas were distinguished from cerebral metastases by peritumoral N-acetylaspartate (NAA)/creatine (Cr), choline (Cho)/Cr, Cho/NAA, and rCBV. There was no significant difference between the two tumor groups in terms of ADC and FA.
Tsuchiya K, et al. ^39^ 2005	Retrospective study	Brain tumors [HGG, SBM]	14	HGG (49), SBM (60)	HGG (17–70 years), SBM (55–70 years)	7	7	Japan	1.5 T	enhancing tumoral, & non-enhancoing peritumoral, normal white matter	FA	HGG [4 GB, 2 AA, and one anaplastic oligodendroglioma], SBMs [four lung cancer,1 colon cancer, and one uterus cancer. The remaining patient’s lesion was adeno- carcinoma of unknown primary]	The FA values of the enhancing and non-enhancing parts did not differ significantly between the two groups. 5 of the seven metastatic patients had subcortical white-matter fibre displacement in the visual evaluation, while just one glioma patient did. Furthermore, 3 of the seven metastasis patients were able to distinguish between tumor and oedema, while none of the glioma patients could. Although FA values are ineffective in distinguishing between the two groups, visual variations in FA values can be used to do so. Another sign of metastasis is the displacement of white-matter filaments.
Lu S, et al. ^40^ 2004	Retrospective study	Brain tumors [GBM, SBM]	20	GBM (51.7 years ± 15.2)), SBM (52.9 years ± 11.0)	17–81 year	10	10	USA	1.5 T	tumoral & peritumoral	FA & MD	SBMs [two metastatic melanomas, one breast carcinoma, five lung carcinomas, and two renal cell carcinomas].	There was no statistically significant difference in peritumoral MD and FA values between intraaxial and extraaxial lesions, or between high- and low-grade gliomas. In the case of intraaxial tumors, the measured mean peritumoral MD of metastatic lesions was 0.733 × 10^3^mm^2^/sec±0.061 (SD), which was considerably greater than that of gliomas, which was 0.587 ± 0.093×10^3^ mm^2^/sec (*p* < 0.05). The Tumor infiltration index (TIIs) of the edema around meningiomas and metastases (mean, 0 ± 35) and the TIIs of the edema surrounding gliomas (mean, 64 ± 59) were similarly statistically significant (*p* < .05).
Law M, et al. ^16^ 2002	Clinical study	Brain tumors [HGG, SBM]	36	51.9 years	15–80 years	24	12	USA	1.5 T	tumoral & peritumoral	rCBV	33 HGGs [28 GBM. 5 AA] 18 SBMs, [2melanomas, and 16 were carcinomas, (two renal, three breast, four lung, two gastric, one mucinous adenocarcinoma from colon, and four from an unknown primary site].	In high-grade gliomas and metastases, the assessed relative cerebral blood volumes in the peritumoral area were 1.31 ± 0.97 (mean ± SD) and 0.39 ± 0.19, respectively. There was a statistically significant difference (*p* < 0.001). Spectroscopic imaging revealed increased choline levels in the peritumoral area of gliomas (choline-to-creatine ratio of 2.28 ± 1.24) but not in metastases (choline-to-creatine ratio of 0.76 ± 0.23). There was a statistically significant difference (*p* = 0.001).
Bulakbasi N., et al. ^41^ 2005	Prospective	Brain tumors [HGG, SBM]	39	39.93 ± 18.33 years	eleven to 85 years	22	17	Turkey	1.5 T	tumoral & peritumoral	rCBV	SBMs (seven breast carcinomas, four lung small-cell carcinomas, three colon mucinous adenocarcinomas, two ovarian adeno- carcinomas, and one squamous cell carcinoma).	The mean differences in rCBVT and rCBVP values between LGGT (2.30 ± 1.12 and 1.18 ± 0.24) and HGGT (5.42 ± 1.52 and 2.17 ± 0.82) (P.001), HGGTs and SBMs (3.21 ± 0.98 and 0.97 ± 0.09) (*p* < .001), and LGGTs and METs (*p* < .05 and *p* < .001, There was no apparent cutoff value. When non-astrocytic glial tumors were eliminated, a clear rCBVT cutoff value of 2.6 was found for distinguishing of low-grade (1.75 ± 0.38; LGA) *vs* high-grade (4.78 ± 0.99; HGA) astrocytomas. The degree of malignancy was linearly associated with the rCBVT levels (*r* = 0.869; *p* < .001). rCBVP cutoff values of 1.1 and 1.2 were shown to be very efficient in distinguishing SBMs from LGGTs and HGGTs, respectively. In both grading and distinction, the overall effectiveness of rCBV was greater.
Shi, L et al. ^42^ 2010	Clinical trial	Brain tumors [Astrocytoma (Anaplastic Astrocytoma glioblastoma, a), SBM, brain abcess ]	43	45.6	13–71	35 (19 anaplastic astrocytoma, 16 glioblastoma)	8	China	1.5 T	Tumoral & peritumoral & normal cerebral white matter	DTI (MD and FA)	Solitary brain lessions	The cavity of a high-grade astrocytoma and brain metastases had hypointense signals, whereas the majority of the brain abscess cavities had high signal intensity, with one instance having inconsistent signal intensity. The measurements of mean diffusivity (MD) and fractional anisotropy (FA) might be utilized to distinguish between a tumor and a brain abscess.

a FA, fractional anisotropy; HGG/HGGs, high-grade glioma/s; ADC, Apparent diffusion coefficient; mean ± SD, means and standard deviation; SBM/SBMs, Solitary brain metastases; PSC, peritumoral signal-change; NA, not available; TBF,. the tumor blood flow; DTI, Diffusion Tensor Imaging; DWI, diffusion weighted imaging; DTI, diffusion tensor imaging; DSCI, dynamic-susceptibility contrast imaging; DSC, dynamic susceptibility contrast; ADC, apparent diffusion coefficient ;DCE, dynamic contrast-enhanced; rPH, relative peak height; rPSR, relative percentage of signal recovery; MRS, Magnetic Resonance Spectroscopy; CBF, Cerebral Blood Flow; T rCBV, Tumoral-relative Cerebral Blood Volume; T rCBF, Tumoral relative Cerebral Blood Flow; T rMTT, Tumoral relative Mean Transit Time; P rCBV / rCBVp*, Peritumoral relative Cerebral Blood Volume; P rCBF, Peritumoral relative Cerebral Blood Flow; P rMTT, Peritumoral relative Mean Transit Time; ROI, regions of interest; IPR, immediate peritumoral region; GB, Glioblastoma; GBM, glioblastoma multiformes; AA, anaplastic astrocytomas; WHO, World Health Organisation; NODDI, neurite orientation dispersion and density imaging; MAP-MRI, mean apparent propagator magnetic resonance imaging; DKI, diffusion kurtosis imaging; PSC, Peritumoral signal change; AUC, areas under curve; NET2, non-enhancing peritumoral T2 hyperintense region* ***; TII/s,*** *Tumor infiltration in*

Table 6 summarizes the characters of the included studies.

#### Intratumoral rCBV

**Figure 6.**
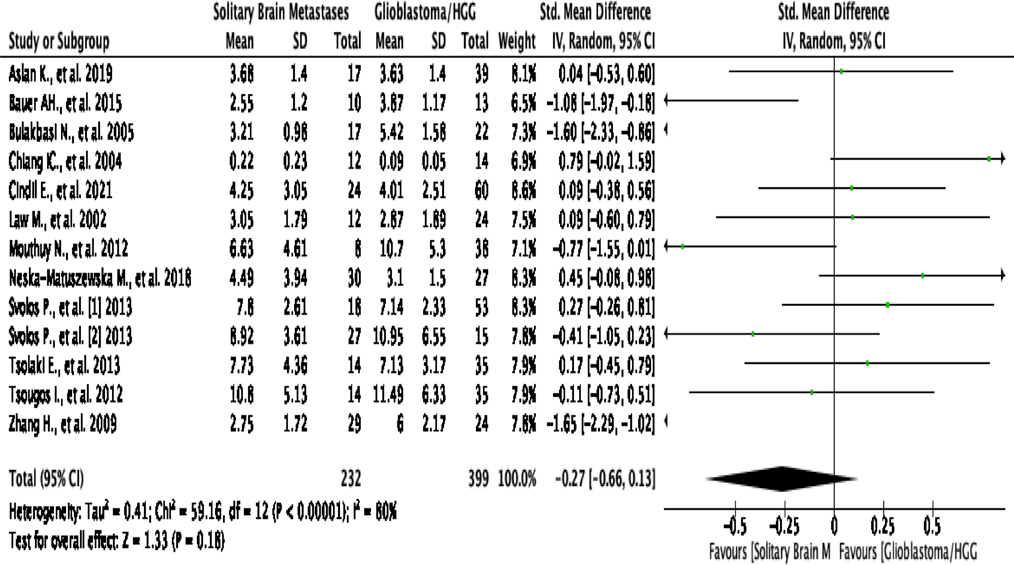
Forest plot standard mean difference of rCBV in intratumoral HGG *vs* MET. HGG, high-grade glioma; rCBV, relative cerebral blood volume.

This analysis shows that pooling data from 13 studies reported value of rCBV in the intratumoral region^2,15,16,25,28,30,31,33–35,37,38,41^ show that HGG patients are no significant difference with SBM/the control group (SMD [95% CI] = −0.27 [-0.66, 0.13]; P for overall effect = 0.18), by a high level of heterogeneity (I^2^  = 80%; *p*  < 0.00001) (Figure 6). An asymmetrical funnel plot was discovered, advising there is publication bias (Figure 1).

#### Peritumoral rCBV

**Figure 7.**
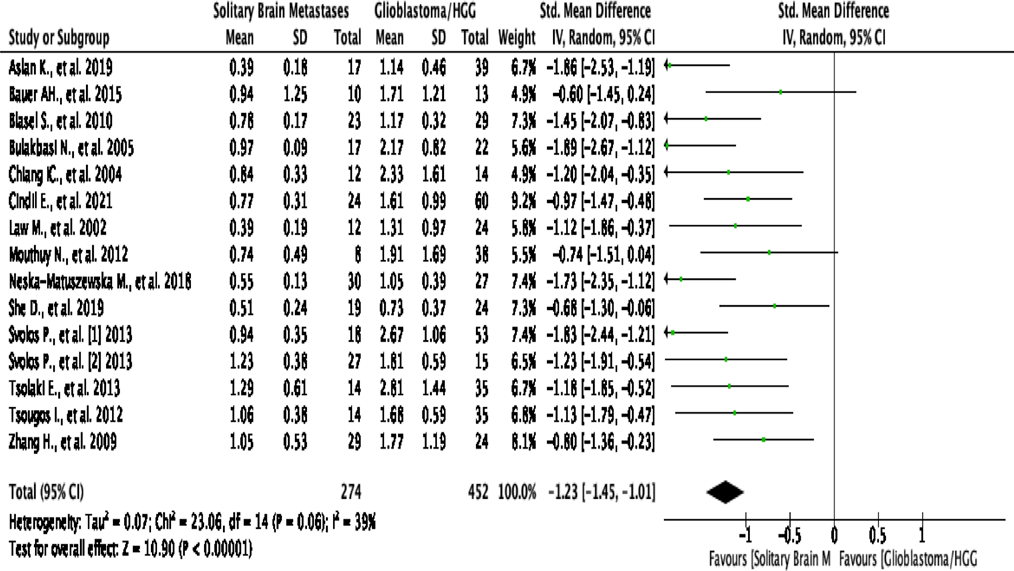
Forest plot standard mean difference of rCBV in peritumoral HGG *vs* MET. HGG, high-grade glioma; rCBV, relative cerebral blood volume.

This analysis shows that pooling data from 15 studies stated estimate of rCBV in the peritumoral edema region^2,7,15,16,25,27,28,30,31,33–35,37,38,41^ show that HGG patients are significantly higher than SBM/the control group (SMD [95% CI] = −1.23 [-1.45 to -1.01]; P for overall effect <0.00001), by a low level of heterogeneity (I^2^ =  39%; *p*  =  0.06) (Figure 7). An asymmetrical funnel plot was realized, suggesting there is publication bias Figure 2.

##### Intratumoral FA

**Figure 8.**
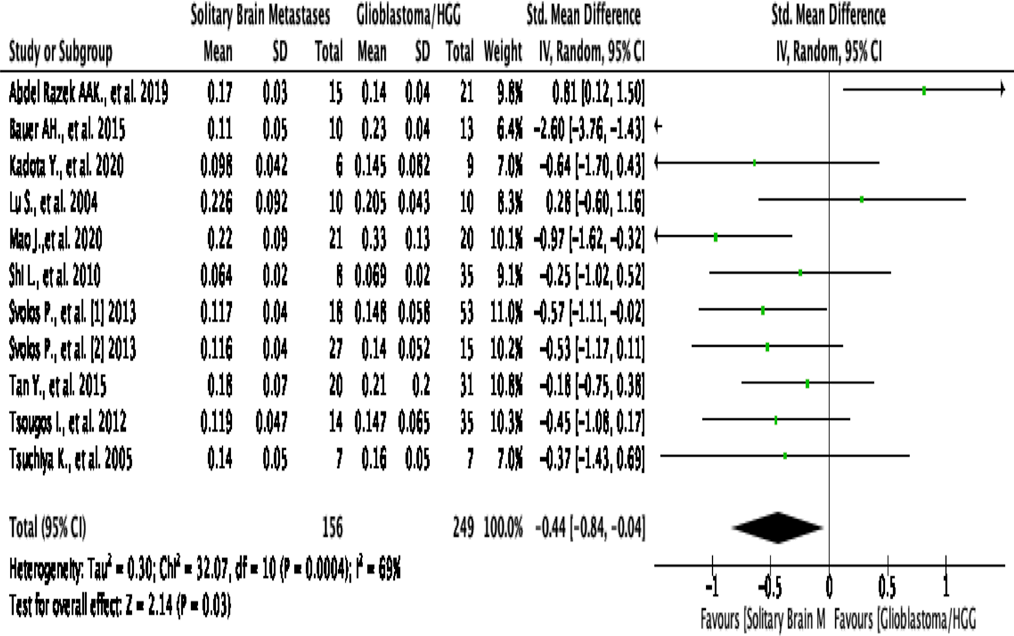
Forest plot standard mean difference of FA in intratumoral HGG *vs* MET. HGG, high-grade glioma; FA, fractional anistrophy.

This analysis shows that pooling data from 11 studies were involved value of FA in the intratumoral region^2,9,26,29,30,32,35,38–40,42^ show that HGG significantly higher than SBM/the control group (SMD [95% CI] = −0.44 [-0.84,–0.04]; P for overall effect = 0.03), by a moderate level of heterogeneity (I^2^ =  69%; *p* = 0.0004) (Figure 8). Asymmetrical funnel plot was obtained, advising a publication bias (Figure 3).

### Peritumoral FA

**Figure 9.**
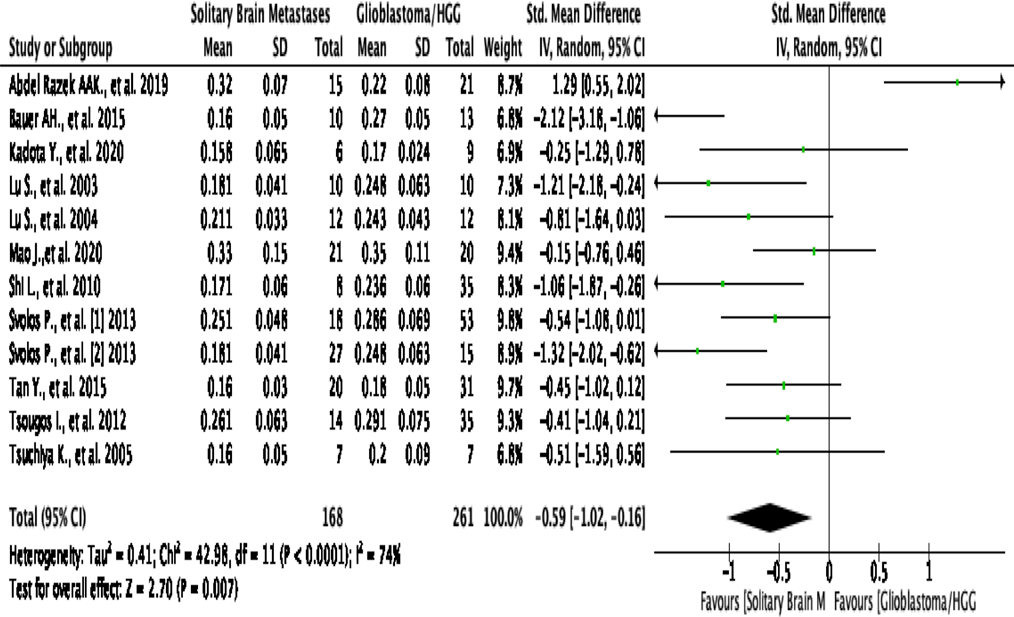
Forest plot standard mean difference of FA in peritumoral HGG *vs* MET. HGG, high-grade glioma; FA, fractional anistrophy.

This analysis shows that pooling data from all 12 studies reported the value of FA in the peritumoral edema region^2,9,26,29,30,32,35,36,38–40,42^ show that HGG patients are significantly higher than SBM/the control group (SMD [95% CI] = −0.59 [-1.02,–0.16]; P for overall effect = 0.007), by a moderate level of heterogeneity (I^2^  = 74%; *p* < 0.0001) (Figure 9). An asymmetrical funnel plot was achieved, advising there is publication bias (Figure 4).

The analysis evaluating the difference between HGG and SBM included 970 patients (597 patients of HGG and 373 controls grouped SBM) into four categories: rCBV in intratumoral (399 patients/HGG and 232 controls/SBM), rCBV in peritumoral (452 patients/HGG and 274 controls/SBM), and FA in intratumoral (249 patients/HGG and 156 controls/SBM), FA in peritumoral (261 patients/HGG and 168 controls/SBM).

The diagnosis of HGG and SBM is primarily established histopathologically, MRI diagnosis evaluating perfusion MR and diffusion MR, cases defined by measurement of rCBV and FA.

### Heterogeneity and publication bias

The funnel plot was used to evaluate the publication bias visually (Figure 1, Figure 2, Figure 3, Figure 4). Visual inspection reveals the distribution of the standard mean difference obtained from the studies included in the pooling analysis of rCBV in intratumoral and peritumoral regions asymmetrical, suggesting publication bias. The pooling analysis of FA in intratumoral and peritumoral regions is asymmetrical, suggesting publication bias. Interstudy heterogeneity was significant among the studies in the intratumoral region (I^2^ = 80 and 69%) and peritumoral region (I^2^ = 39 and 74%). Whereas the I^2^ for the rCBV in the intratumoral subgroup was 80%, I^2^ for the rCBV in the peritumoral subgroup was 39%, and I^2^ for the FA in the intratumoral subgroup was 69%, and I^2^ for the FA in peritumoral subgroup was 74%.

## Discussion

Based on this study’s outcomes, we found that patients with HGG have significantly higher rCBV and FA in the peritumoral edema region and higher FA in the intratumoral region compared to the SBM/control groups. However, no significant difference was identified between HGGs and SBMs when pooling rCBV data in the intratumoral region. Up to the authors’ knowledge, we specify how HGGs and SBMs can differ in diagnosing. Checking that the data extracted from the trial reports are correct, conducting subgroup analysis, choosing the random effect model, and excluding studies were performed to address the heterogeneity. The predefined accurate search criteria, and precise selection and evaluation of methodological quality for included studies, strengthen this study.

Calculating the CBV in the peritumoral edema makes it feasible to distinguish glioblastomas from metastases.^46^ However, most researchers have found that rCBV in intratumoral regions cannot consistently discriminate between these two conditions^2,16,47,48^ owing to the gap seeping from tumor arteries that caused an incorrect CBV estimation.^6,45,49^ Utilizing DSC perfusion imaging to quantify rCBV in increasing tumor volumes did not assist in differentiating these two tumors.^14–16,38,50^

The importance of FA in distinguishing HGGs and metastases inside the intratumoral section and the peritumoral edema yielded mixed findings. Some showed that FA in the intratumoral is higher in glioblastomas than metastases, an attribute to the fact that glioblastomas are often more cellular than brain metastases.^51^ While others showed no significant differences,^52^ the outcome may be described by diverse grades of tumor infiltration in these two tumor types, with FA being mostly influenced by tumor infiltration. However, some showed FA in peritumoral significantly higher in metastases than HGG,^51^ and others showed significantly lower in metastases than HGG.^52^ The peritumoral edema of the metastasis shows different regions of variable compressed, displaced, and edematous tracts, and the values differ in each region.^53^ The shortage of standardized methods with regards to selection, capture, and post-processing of ROI is one likely reason for these contradictory results.

As a result, we anticipated that HGG perfusion characteristics in intratumoral may not differ from SBM. However, perfusion in the peritumoral region and diffusion in intratumoral and peritumoral regions can differ from brain metastasis in this study. We also suggested that FA and rCBV parameters derived in these tumor subregions may be utilized to differentiate between the two tumor types. The optimum model for identifying these two tumors was built to attain this goal by integrating perfusion imaging technique (DSC metrics) from peritumoral regions and diffusion imaging method (DTI metrics) in intratumoral and peritumoral.

The current body of knowledge regarding the imaging differentiation of solitary metastasis and HGG is using rCBV in DSC. Other diffusion imaging methods, NODDI and DTI, hold promise for accurate distinction in the future. The development of several radiomics and machine-based learning algorithms is also ongoing. To attain high levels of accuracy, several sophisticated imaging modalities were frequently combined. Further primary studies in combining perfusion of rCBV with diffusion of FA, in the peritumoral and intratumoral area is required to add evidence to support our findings.

Other perfusion imaging methods to differentiate these two conditions are dynamic contrast-enhanced magnetic resonance perfusion (DCE); and arterial spin labeling (ASL).

DCE offers details regarding tissue characteristics at the microvascular level just like DSC does in general. It appears that few studies have used DCE alone to distinguish between glioblastoma and brain metastases. But when employed as an additional imaging modality, DCE may provide a more thorough evaluation of brain tumor angiogenesis than DSC due to its capacity to investigate the blood–brain barrier and vascular permeability quantitatively..^54^

Dynamic susceptibility contrast-enhanced perfusion (DSC) is one of the perfusion imaging techniques. The rCBV obtained from DSC, which has 96% (88–100%) specificity, 90.20 (23.10–352.27) DOR, and 82% (72–90%) sensitivity in the investigation, is confirmed to have an excellent diagnostic value for distinguishing HGGs from intracranial metastases.^55^ DSC does have several drawbacks, *i.e.* it may contain artifacts from surgical gear or bone-air contacts close to the base of the skull.^54^

A further perfusion imaging method called ASL uses electromagnetically marked arterial blood water as an intrinsic tracer that may be utilized to measure cerebral blood flow (CBF) in tumors. Only a few research have looked at the clinical efficacy of ASL to distinguish GBM from brain metastasis, despite its clinical value and suitability for the characterization of brain malignancies. However, there is a significant overlap between GBM and brain metastases regarding the qualitative and quantitative ASL characteristics. According to recent research by Bauer et al, the distinction between GBM and SBM may be made with 98% accuracy using a combination of DWI, DSC perfusion, and DCE perfusion MR measures in the peritumoral T2 hyperintensity region. This should only be used with care because of the limits of ASL and the relatively low interobserver agreement. Further investigation into the causes and potential solutions for this interobserver variability would be beneficial. Furthermore, there are strong correlations between DSC-CBV and ASL-CBF in comparative studies of the two techniques for assessing brain tumors. According to one of these investigations, the susceptibility artifact in the tumor region or peritumoral area is less on ASL pictures than on DSC images.

A part to DTI, other diffusion imaging methods to differentiate these two conditions are DWI measurement of apparent diffusion coefficient (ADC); and neurite orientation dispersion and density imaging (NODDI).^54^

Water diffusion in tissue is measured by the ADC. Applying theoretical mathematical formulae with variables like the strength of the magnetic field, beginning signal intensity, and post-imaging signal intensity can yield ADC values for numerous DW pictures. Theoretically, DWI may be used to create models that analyze the cellularity of cerebral lesions, which would help differentiate them. However, one research uses 3 Tesla MRI technology at a level that demonstrates statistical significance to show that tumor ADC levels in malignant gliomas are distinct from those in metastases. According to several theories, a decrease in ADC values during imaging suggests increased cellularity, which might be a valuable indicator of whether or not tumor cells have invaded the nearby tissues. Several investigations support this by comparing the peritumoral edema of metastases and HGGs. Although ADC values may be calculated using DWI, this model is simplistic since it assumes isotropic water diffusion (*i.e.* the same in all directions).^54^

On a regular MRI scanner, NODDI is an efficient diffusion MRI method that may be used to determine how complex neurites are *in vivo*. It is possible to map the distribution and density of neurites inside brain tissue, which is helpful for understanding how the brain is connected. For instance, it can reveal information about other disease pathologies, such as gliomas or brain metastases.^54^

The intracellular space, extracellular space, and cerebrospinal fluid are the three compartments identified by NODDI as constituting each voxel’s simplified brain architecture. In contrast to DTI analysis alone, NODDI can offer more precise information on the microstructural alterations of neurites. By creating intracellular volume fraction (VIC), isotropic volume fraction (VISO), and extracellular volume fraction (VEC), NODDI provides a compartment map as opposed to DTI, which uses indices like FA to map out water transport within areas of interest.^54^

Kadota et al discovered that, when compared to FA, VIC, and VISO, VEC in the peritumoral signal change region was most helpful in separating glioblastoma from metastases. With a threshold value of 0.48, they discovered that VEC offered 100% sensitivity and 83.3% specificity. Mao et al recently assessed the performance of five diffusion-weighted MRI models for separating HGG from metastases. They discovered that NODDI performed better than DTI and DWI in separating HGG from metastases. VISO was the most effective measure for differentiating between the two.^54^

Overall, the MR techniques presented here lead to a tremendous increase of knowledge that can be obtained during an MRI session in addition to conventional structural MRI, and are obviously a great asset to making the final diagnosis or providing better differentials.^56^

## Limitations

Although this meta-analysis included a wide variety of publications in the search process, publication bias can occur inevitably. We may not cover all the studies with data relevant to our study due to difficulty in obtaining positive findings indexed.

The constraint of this study that might limit the confidence level of our findings was: the ability to verify pooling data from studies due to various diagnostic techniques and cut-off values; tumors of varying sizes and locations; diversity of the population, study design, diagnostic and examination criteria contributed to heterogeneity between study.

## Conclusion and recommendations

Our findings from this meta-analysis showed the outcomes support to use of rCBV and FA in the peritumoral region and FA in intratumoral region measurement for differentiating the HGG and SBM. Our study is the first meta-analysis examining a combination of MR perfusion value of rCBV and MR diffusion value of FA parameters to construct a predictive multiparametric imaging approach study on the differentiation between HGG and SBM using MRI that we are aware.

We recommend that healthcare professionals study the capacity difference between HGGs and SBMs when assessing patients with intracranial/brain tumors. Furthermore, we recommend that researchers conduct: advanced studies to improve the diagnostic methods, other MRI techniques to increase diagnostic values, the diagnostic performance of perfusion MR and diffusion MR, and discover more diagnostic tools.

## Supplementary Material

bjr.20220052.suppl-01
