# Supplementary material for: Differentiation of high grade glioma and solitary brain metastases by measuring relative cerebral blood volume and fractional anisotropy: a systematic review and meta-analysis of MRI diagnostic test accuracy studies
Source: Br J Radiol. 2022 Dec 8;96(1141):20220052. doi: 10.1259/bjr.20220052 (PMC10997014; doi:10.1259/bjr.20220052)
Supplement: bjr.20220052.suppl-01 [file bjr.20220052.suppl-01.docx]

**Supplementary Content**

Differentiation of High Grade Glioma and Solitary Brain Metastases by Measuring of Relative Cerebral Blood Volume and Fractional Anisotropy: A Systematic Review and Meta-Analysis

1. Appendix A: Search strategy used in the current systematic review and meta- analysis.

### Appendix B: Abbreviations

1. **Appendix A: Search strategy used in the current systematic review and meta- analysis.**
2. using pubmed search engine keywords with syntax MeSH and text word "discriminat” OR "different" OR "distinguish" OR "diagnosis, differential AND "glioblastoma" OR "gbm" OR "gb" OR "astrocyt" OR "glioma" OR "gliosarcom" OR "glioblastoma multiforme" OR "multifocal glioblastoma" OR "multicentric glioblastoma" OR "Grade IV astrocytoma" OR "giant cell glioblastoma" OR "glioblastoma" AND "solitair" OR "solitary" OR "single" AND "brain" OR "central nervous sys" OR "encephalon" OR "cerebral" OR "intracranial" OR "intracerebral" AND "metastasis" OR "cancer" OR "tumor" OR "tumour" OR "neoplas" OR "carcinoma" OR "malignan" OR "neoplasm metastasis" AND "relative cerebral blood volume" OR "rCBV" OR "cerebrovascular circulation" OR "cerebral circulat" OR "brain blood flow" OR "cerebral blood flow" OR "cerebral perfusion pressure" OR "cerebral blood volume" OR "cerebrovascular circulation" OR "Diffusion MRI" OR "diffusion tensor imaging" OR "fractional anisotropy" OR "FA" OR "mean diffusivity" OR "Diffusion Magnetic Resonance Imaging" OR "diffusion tensor imaging" AND ((humans[Filter]) AND (english[Filter])), Search results: 234 studies, Filters applied : humans, English get 194 studies. Date of search 14^th^September 2021;
3. using Scopus search engine, used the following search keywords:( different* OR discriminat* OR distinguish OR distinct* ) AND  ( glioblastoma* OR gbm OR gb OR astrocyt* OR gliom* OR gliosarcom* OR "glioblastoma multiforme" OR "multifocal glioblastoma" OR "multicentric glioblastoma" OR "grade iv astrocytoma" OR "giant cell glioblastoma" ) AND  ( "solitary brain metast*" OR "solitair* brain metasta*" OR "single brain metasta*" OR "neoplasm metasta*" OR "tumor metasta*" OR "cns metas*" OR "central nervous system metast*" OR tumor ) AND ( "relative cerebral blood volume" OR rcbv OR "cerebral blood volume" OR "fractional anisotropy" OR "mean diffusivity" ) . Search result: 683 studies, filter get: 535 results of articles only, journal, human, English. Date of search 14^th^September 2021;
4. using WOS search engine keywords #1 (different* OR discriminat* OR distinguish OR distinct*), #2 glioblastoma* OR GBM OR GB OR astrocyt* OR gliom* OR gliosarcom* OR "glioblastoma multiforme" OR "multifocal glioblastoma" OR "multicentric glioblastoma" OR "Grade IV astrocytoma" OR "giant cell glioblastoma"), #3 solitair* OR solitary* OR single), #4 brain OR "central nervous sys*"OR CNS OR encephalon OR cerebral OR intracranial OR intracerebral), #5 metastasis* OR cancer* OR tumor* OR tumor OR neoplas*OR carcinoma* OR malignan*), #6 (#3 AND #4 AND #5), #7 ("relative cerebral blood volume" OR rCBV OR "cerebrovascular circulation" OR "cerebral circulat*" OR "brain blood flow" OR "cerebral blood flow" OR "cerebral perfusion pressure"), #8 ("Diffusion MRI" OR "Diffusion tensor imaging" OR "fractional anisotropy" OR “mean diffusivity” OR MD OR FA ), #9 (#7 AND #8), #10 (#1 AND #2 AND #6 AND #9). Search result: 339 studies, after filter English, articles, get 310 studies. Date of search 14^th^September 2021;
5. using Cochrane library search engine keywords different* OR discriminat* OR distinguish OR distinct* AND glioblastoma* OR GBM* OR GB* OR astrocyt* OR glioma* OR gliosarcom* OR "glioblastoma multiforme" OR "multifocal glioblastoma" OR "multicentric glioblastoma" OR "Grade IV astrocytoma" OR "giant cell glioblastoma*" AND solitair* OR solitary* OR single AND (brain OR "central nervous sys*"OR CNS OR encephalon OR cerebral OR intracranial OR intracerebral) AND metastasis* OR cancer* OR tumor* OR tumor OR neoplas*OR carcinoma* OR malignan* AND ("relative cerebral blood volume" OR rCBV OR "cerebrovascular circulation" OR "cerebral circulat*" OR "cerebral perfusion") OR ("cerebral diffusion" OR “fractional anisotropy” OR “mean diffusivity”). Search result: 17 trial studies and 1 cochrane review, after manually exclude the review study get 17 studies. Date of search 14^th^September 2021.

### II. Appendix B: Abbreviations

*1. FA, fractional anisotropy;*

*2. HGG/HGGs, high-grade glioma/s;*

*3. ADC,Apparent diffusion coefficient;*

*4. mean±SD, means and standard deviation;*

*5. SBM/SBMs, Solitary brain metastases;*

*6. PSC, peritumoral signal-change;*

*7. NA, not available;*

*8. TBF,. the tumor blood flow;*

*9. DTI, Diffusion Tensor Imaging;*

10. *DWI, diffusion weighted imaging;*

*11. DTI, diffusion tensor imaging;*

*12. DSCI, dynamic-susceptibility contrast imaging;*

*13. DSC, dynamic susceptibility contrast;*

*14. ADC, apparent diffusion coefficient ;*

*15. DCE, dynamic contrast-enhanced;*

*16. rPH, relative peak height;*

*17. rPSR, relative percentage of signal recovery;*

*18. MRS, Magnetic Resonance Spectroscopy;*

*19. CBF, Cerebral Blood Flow;*

*20. T rCBV, Tumoral-relative Cerebral Blood Volume;*

*21. T rCBF, Tumoral relative Cerebral Blood Flow;*

*22. T rMTT, Tumoral relative Mean Transit Time;*

*23. P rCBV /* rCBVp*, Peritumoral relative Cerebral Blood Volume;*

*24. P rCBF , Peritumoral relative Cerebral Blood Flow;*

*25. P rMTT, Peritumoral relative Mean Transit Time;*

*26. ROI, regions of interest;*

*27. IPR, immediate peritumoral region;*

28. *GB, Glioblastoma;*

*29. GBM, glioblastoma multiformes;*

*30. AA, anaplastic astrocytomas;*

31. *WHO, World Health Organisation;*

*32. NODDI, neurite orientation dispersion and density imaging;*

*33. MAP-MRI , mean apparent propagator magnetic resonance imaging;*

*34. DKI, diffusion kurtosis imaging;*

*35. PSC, Peritumoral signal change;*

36. *AUC, areas under curve;*

*37. NET2, non-enhancing peritumoral T2 hyperintense region;*

*38. TII/s, Tumor infiltration inde*
